# Neural electrophysiological correlates of detection and identification awareness

**DOI:** 10.3758/s13415-023-01120-5

**Published:** 2023-09-01

**Authors:** Stefan Wiens, Annika Andersson, Josef Gravenfors

**Affiliations:** 1 Department of Psychology, Stockholm University, Stockholm, Sweden; 2 School of Bioscience, University of Skövde, Skövde, Sweden

**Keywords:** Consciousness, Neural correlates, ERP

## Abstract

Humans have conscious experiences of the events in their environment. Previous research from electroencephalography (EEG) has shown visual awareness negativity (VAN) at about 200 ms to be a neural correlate of consciousness (NCC). However, when considering VAN as an NCC, it is important to explore which particular experiences are associated with VAN. Recent research proposes that VAN is an NCC of lower-level experiences (detection) rather than higher-level experiences (identification). However, previous results are mixed and have several limitations. In the present study, the stimulus was a ring with a Gabor patch tilting either left or right. On each trial, subjects rated their awareness on a three-level perceptual awareness scale that captured both detection (something vs. nothing) and identification (identification vs. something). Separate staircases were used to adjust stimulus opacity to the detection threshold and the identification threshold. Bayesian linear mixed models provided extreme evidence (BF10 = 131) that VAN was stronger at the detection threshold than at the identification threshold. Mean VAN decreased from $-$2.12 microV [$-$2.86, $-$1.42] at detection to $-$0.46 microV [$-$0.79, $-$0.11] at identification. These results strongly support the claim that VAN is an NCC of lower-level experiences of seeing something rather than of higher-level experiences of specific properties of the stimuli. Thus, results are consistent with recurrent processing theory in that phenomenal visual consciousness is reflected by VAN. Further, results emphasize that it is important to consider the level of experience when searching for NCC.

## Introduction

As humans, we have conscious experiences of external events. For example, when an image flashes on a computer screen, we might have a lower-level experience that something was there (detection) or a higher-level experience of what it was (identification). To determine the neural mechanisms of these experiences of content (Aru & Bachmann, 2017), research has focused on finding the neural correlates of consciousness (NCC): neural activity that is consistently associated with particular experiences (Crick & Koch, 1990). Because electrocortical (EEG) activity is relatively easy to record and has excellent time resolution (Biasiucci et al., 2019; Luck, 2014), it has been used widely to study NCC. In vision, two event-related potentials (ERPs) derived from EEG have been suggested as NCC of content: visual awareness negativity (VAN) at about 200 ms, and late positivity (LP) after about 300 ms (Förster et al., 2020). Similar EEG correlates have been observed in other modalities such as hearing (Eklund & Wiens, 2019) and touch (Auksztulewicz & Blankenburg, 2013). Because the early negativity (e.g., VAN in vision) is generated in sensory cortices (Meyer, 2011; Snyder et al., 2015), its topography varies with modality (Dembski et al., 2021).

The timing difference between an early process (VAN) and a late process (LP) is important for two prominent theories of consciousness: recurrent processing theory and global neuronal workspace theory. Recurrent processing theory stipulates that conscious experiences are mediated by early, recurrent processing in sensory areas (Lamme, 2006, 2010). Because VAN is believed to capture early, recurrent processing, it is considered to be an NCC (Eklund & Wiens, 2018; Förster et al., 2020; Lamme, 2018). In contrast, global neuronal workspace theory argues that conscious experience is elicited only after activity from sensory modalities is propagated to areas in the global workspace that involve reporting, memory formation, and other cognitive processes (Dehaene & Changeux, 2011; Dehaene et al., 2006; Mashour et al., 2020). Because these ignition processes occur late, VAN is only a correlate of preconscious processes whereas LP is an NCC (Lamy et al., 2009; Salti et al., 2012).

The debate about VAN and LP as NCC has been going on for several decades (Förster et al., 2020; Koivisto & Revonsuo, 2010). In the last decade, however, evidence has accumulated against the idea of LP as an NCC. This evidence demonstrates that in contrast to VAN, LP lacks sensitivity: LP is absent even though subjects report being aware of the stimuli (Dembski et al., 2021; Koivisto & Revonsuo, 2010). In vision, these studies used different designs such as inattentional blindness (Dellert et al., 2021; Pitts et al., 2012, 2014; Schlossmacher et al., 2020), attentional blink (Dellert et al., 2022), and no-report tasks (Cohen et al., 2020; Kronemer et al., 2022). For example, in a classic study with inattentional blindness, Pitts et al. (2014) showed that even though subjects were aware of the stimuli, LP was observed only when the stimuli were task relevant and not when they were task irrelevant. Similarly, in no-report tasks, LP was absent when subjects did not have to report on their awareness of visual stimuli (Kronemer et al., 2022). In sum, because of the lack of sensitivity, LP cannot be considered an NCC, thus refuting the claim of global neuronal workspace theory. Instead, LP seems to reflect post-perceptual processes related to the experimental task, such as the preparation for a report or the detection of a stimuli as task-relevant (Dembski et al., 2021; Förster et al., 2020; Koivisto & Revonsuo, 2010). Because LP shows a close relationship with post-perceptual processes, we discuss LP as well as VAN in the present study.

When examining NCC of content, it is important to explore whether a particular EEG response (e.g., VAN) is associated with particular experiences (Aru & Bachmann, 2017; Koivisto et al., 2017). A common measure of different experiences of content is the perceptual awareness scale (PAS) (Sandberg & Overgaard, 2015). This scale was developed with the following assumption: Because experiences differ depending on stimuli and task, it is critical to interview subjects about their experiences and to develop the rating scale in close cooperation with the subjects (Sandberg et al., 2013; Sandberg & Overgaard, 2015). In the original study (Ramsøy & Overgaard, 2004), subjects were shown masked visual stimuli that differed in location, color, and shape. Interviews of the subjects revealed that in general, subjects described their experiences in terms of four levels: nothing (PAS1), a brief glimpse (PAS2), an almost clear experience (PAS3), or a clear experience (PAS4). These labels summarized more elaborate descriptions of each level of experience. For example, the description of PAS3 referred to a feeling of almost being certain in one’s answer. However, this reference to other psychological processes (e.g., certainty) was included only because subjects found these helpful when describing their conscious experiences. Because these labels may not be adequate for other stimuli and tasks, it is recommended to adjust the number of PAS levels and the meaning and description of each level in a pilot study (Sandberg et al., 2013; Sandberg & Overgaard, 2015).

In a recent study, Koivisto et al., 2017 claimed that VAN is a correlate of lower-level experiences rather than of higher-level experiences. Lower-level experiences refer to elementary phenomenal experiences of seeing something, of the presence of visual sensations that do not involve higher-level operations such as categorization. These experiences may be described as conscious detection. Higher-level experiences refer to experiences of the specific properties of the stimulus. These experiences involve higher-level operations such as categorization and labeling. These experiences may be described as conscious identification. To examine their claim, Koivisto et al. (2017) recorded EEG while subjects were shown single digits (from the set 3, 4, 6, and 7) in the middle of the screen. On separate blocks, subjects performed two tasks: a detection task and a classification task. In the detection task, subjects had to respond whether a stimulus (digit) was presented. In the classification task, subjects had to indicate whether the digit was smaller or larger than 5. On each trial, subjects rated their conscious experience of the digit on a modified version of the original four-level PAS (Ramsøy & Overgaard, 2004). The main changes were that PAS2 was defined as "I saw something (but could not identify the stimulus)" and PAS3 was defined as "I saw the stimulus almost clearly (and could identify it)." For each subject, the contrast and the duration of the digits were adjusted to two awareness thresholds. The *detection threshold* was targeted in the detection task and compared trials rated as PAS2 with trials rated as PAS1 (i.e., PAS2 − PAS1). Thus, the detection threshold captured lower-level experiences. The *identification threshold* was targeted in the classification task and compared PAS3 trials with PAS2 trials (i.e., PAS3 − PAS2). Thus, the identification threshold captured higher-level experiences.

For VAN-relevant mean amplitudes, results showed a statistically significant interaction between awareness and task (Koivisto et al., 2017). Follow-up analyses showed that for the detection task, mean amplitudes at the VAN-relevant interval were more negative to aware trials (PAS2) than unaware trials (PAS1), $p\, {=}\, .014$; this finding provides evidence for VAN. In contrast, for the identification task, mean amplitudes did not differ significantly between aware trials (PAS3) and unaware trials (PAS2), $p\, {=}\, .562$. For LP-relevant amplitudes, mean amplitudes across tasks were more positive for aware than unaware trials; this finding provides evidence for LP. Results did not suggest that LP differed by task. The authors concluded that "the elementary phenomenal experiences of ’seeing something,’ without awareness of the higher properties of the stimulus, had a unique correlate (VAN) that was not present at the identification threshold" (p. 1628). Thus, Koivisto et al., 2017 claimed that VAN is an NCC of lower-level experiences (detection) rather than higher-level experiences (identification).

When discussing this claim (Koivisto et al., 2017), we propose that it is useful to consider two possible interpretations: A *strong* version is that there is no VAN for higher-level experiences (identification). A *weak* version is that VAN is larger (more negative) for lower-level experiences (detection) than for higher-level experiences (identification). The weak claim implies only that VAN is less sensitive to identification than detection. Accordingly, VAN should be smaller for identification than detection.

Although the study by Koivisto et al. (2017) is important, it has two main limitations. First, the two threshold conditions were obtained during different tasks with different instructions. Thus, thresholds were perfectly correlated with task instructions. In support of the idea that task differences may have affected results, a supplementary analysis showed that LP at the detection threshold (PAS2 − PAS1) was larger for the detection task than the identification task (Koivisto et al., 2017). Thus, LP varied by task for identical stimuli at the same threshold. Because task differences per se can affect VAN and LP (Andersen et al., 2022; Jimenez et al., 2020; Windey et al., 2014), it is unclear whether the main findings for VAN and LP (Koivisto et al., 2017) were caused by differences in threshold, differences in task, or both. Second, the claim that VAN is not an NCC of identification is an attempt to prove the null hypothesis (i.e., VAN = 0), but this approach is inherently difficult to do with null hypothesis significance testing (Dienes, 2016; Wasserstein & Lazar, 2016). For example, because sample size was small (*N* = 12), null effects might be expected because of low statistical power (Makin & Orban de Xivry, 2019).

Results of other EEG studies are relevant to examine the claim that VAN is an NCC of lower-level experiences rather than higher-level experiences (Derda et al., 2019; Jimenez et al., 2021, 2018; Tagliabue et al., 2016). In our review of these studies, we focus on whether findings were statistically significant. A non-significant finding is inherently problematic because it may simply be caused by low statistical power (Makin & Orban de Xivry, 2019; Wasserstein & Lazar, 2016). Also, counting significance is not a valid meta-analytic procedure (Borenstein et al., 2009). Nonetheless, we argue that it is informative in the present context. If several studies found a (statistically) significant VAN for the identification threshold, then the strong interpretation is unlikely to be correct. Also, if several studies found that the VAN is (statistically) smaller at the identification threshold than the detection threshold, then the weak interpretation would be supported.

Tagliabue et al. (2016) presented light and dark gratings in the upper right visual field. On each trial, subjects rated whether the grating was light or dark, and also rated their awareness on the original PAS. Linear trend analyses over PAS (i.e., PAS1 to PAS4) showed that VAN-relevant amplitudes became increasingly more negative; this suggests that VAN increased gradually with awareness. This finding is not consistent with the strong interpretation because VAN should not increase from PAS2 to PAS3 (and to PAS4). With regard to the weak interpretation, no analysis examined whether the difference of PAS2 minus PAS1 was larger than the difference of PAS3 minus PAS2. Results for LP-relevant amplitudes showed that amplitudes increased positively with PAS.

The design of the remaining EEG studies (Derda et al., 2019; Jimenez et al., 2018, 2021) was informed by levels of processing (LoP) theory (Jimenez et al., 2020; Windey et al., 2014). According to LoP theory, awareness is gradual for a low-level stimulus/task (e.g., awareness of energy and features), and dichotomous for a high-level stimulus/task (e.g., awareness of the meaning of digits and letters). To examine this theory, the stimuli in the EEG studies combined low-level features (e.g., colors) and high-level features (e.g., digits). On separate tasks, subjects focused on either the low-level features during a low-level task (e.g., color discrimination) or the high-level features during a high-level task (e.g., digit identification). On each trial, subjects rated their awareness on the original PAS (Ramsøy & Overgaard, 2004). From our reading of Koivisto et al. (2017), results of these studies are relevant here because the low-level stimulus/task and the high-level stimulus/task can be viewed as separate tests of the claim by Koivisto et al. (2017). That is, if subjects perform a color discrimination task (low level), lower-level experiences would be captured when subjects report that they can consciously detect the stimuli without awareness of the color (detection), and higher-level experiences would be captured when subjects report that they can consciously identify the colors (identification). Similarly, if subjects perform a digit identification task, lower-level experiences would be captured when subjects report that they can consciously detect the stimuli without awareness of the digit (detection), and higher-level experiences would be captured when subjects report that they can consciously identify the digits (identification).

Jimenez et al. (2018) presented backward-masked digits and letters in different visual quadrants. Subjects performed two separate tasks: a low-level detection task on stimulus location and a high-level identification task on the digits/letters. In both tasks, subjects also rated their awareness on the original PAS. Because of a lack of *clear* (PAS4) trials, these trials were combined with *almost clear* (PAS3) trials. A (statistically) significant VAN was observed for the difference of *weak* (PAS2) minus *nothing* (PAS1) in the detection task and in the identification task, supporting that VAN is an NCC of detection. No significant VAN was observed for the difference of PAS3/4 minus PAS2 in the identification task, consistent with the strong interpretation. However, a significant VAN was also obtained for the difference of PAS3/4 minus PAS2 in the detection task. This later finding does not support the strong interpretation (Koivisto et al., 2017). With regard to the weak interpretation, no analysis explicitly tested whether VAN is larger for the difference of PAS2 minus PAS1 than for the difference of PAS3/4 minus PAS2. For LP-relevant mean amplitudes, results across tasks showed that amplitudes were more positive for PAS3 than PAS1, providing evidence for LP. It was unclear whether PAS2 differed from the other ratings. Overall, LP did not vary with task, similar to results reported by Koivisto et al. (2017).

In a follow-up study (Jimenez et al., 2021), subjects viewed backward-masked, colored line drawings of objects and animals at fixation. Subjects performed either a low-level color discrimination task (blue or red) or a high-level categorization task (object or animal). The original PAS was used, but subjects were instructed to use PAS with regard to either color or category. Because PAS4 was seldom used, PAS4 trials were combined with PAS3 trials. Only the color task showed significant results: A significant VAN for the difference of PAS2 minus PAS1 and the difference of PAS3/4 minus PAS1; PAS3/4 did not differ significantly from PAS2. These findings of null effects for the difference of PAS3/4 minus PAS2 on the color task and the categorization task are consistent with the strong interpretation. LP was observed for the difference of PAS3 minus PAS1, providing evidence for LP. Because this effect was significant only for the category task, results for LP are only partly consistent with previous findings (Jimenez et al., 2018; Koivisto et al., 2017).

In another study (Derda et al., 2019), backward-masked, colored digits were shown at the center of the screen, and subjects performed either a color discrimination task (low level) or a magnitude judgment task on the digits (high level). The original PAS was used, but PAS4 was excluded because of a lack of trials. Results suggested that VAN-relevant amplitudes showed a linear increase in negativity over PAS1 to PAS3, and this linear increase did not differ by task. Thus, results suggested that VAN increased with awareness. This effect is not consistent with the strong interpretation because VAN should not increase from PAS2 to PAS3. With regard to the weak interpretation, the difference of PAS2 minus PAS1 was not significantly larger than the difference of PAS3 minus PAS2. Results for LP-relevant amplitudes showed an interaction of PAS and task. LP was observed only for the high-level stimulus/task.

Taken together, the combined results do not consistently support the claim that VAN is an NCC of lower-level experiences (detection) rather than higher-level experiences (identification) (Derda et al., 2019; Jimenez et al., 2018; Koivisto et al., 2017; Tagliabue et al., 2016). With regard to the strong interpretation, Jimenez et al. (2018) found a significant VAN for the difference of PAS3 (or PAS3/4) minus PAS2 in a low-level detection task, and two other studies found that VAN increased linearly over PAS in a low-level detection task (Derda et al., 2019; Tagliabue et al., 2016) and in a high-level identification task (Derda et al., 2019). With regard to the weak interpretation, results are unclear because only a single study tested formally whether the difference between PAS2 and PAS1 differed from the difference between PAS3/4 and PAS2 (Derda et al., 2019). Because nonsignificant results were obtained, there is currently no evidence for (or against) the weak interpretation.

Inconsistent results may be caused by differences in experimental design. First, it is unclear whether any previous study is a valid test of either version of the claim (Koivisto et al., 2017). Many studies used the original PAS (Ramsøy & Overgaard, 2004), but subjects may not necessarily interpret the difference between PAS2 and PAS3 (weak vs. almost clear) or between PAS3 and PAS4 (almost clear vs. clear) to refer to their awareness of the identity of the stimulus. Therefore, the difference of PAS3 minus PAS2 (or PAS4 minus PAS3) may not necessarily capture an identification threshold unless subjects are explicitly instructed to rate accordingly (Koivisto et al., 2017). Second, it may be difficult to compare previous studies that used widely different stimuli (e.g., digits vs. gratings) and manipulations of visibility (e.g., low contrast vs. backward masking), particularly so because distributions of awareness ratings vary strongly with the stimulus and the manipulations of visibility (Kiefer & Kammer, 2017). Third, studies varied in whether the stimuli were presented in the middle of the screen or in the periphery. Compared to central stimulation, peripheral stimulation leads to more variable awareness ratings, but this variability in awareness is probably caused by differences in attention (Koivisto et al., 2009). Thus, a preferable design is to present stimuli in the middle of the screen to reduce confounding effects of attention.

The goal of the present study was to examine the claim that VAN is an NCC of lower-level experiences rather than higher-level experiences (Koivisto et al., 2017). In the present study, subjects used a three-level PAS to rate their awareness of a ring that tilted either left or right. Lower-level experiences were measured in a detection task and higher-level experiences were measured in an identification task. Below, we refer to lower-level experiences as *detection* and higher-level experiences as *identification*. These terms are used only as convenient labels, as experiences may be conceptualized differently for other stimuli and tasks. For example, lower-level experiences may refer to awareness of the location of stimuli on the screen, and higher-level experiences may refer to awareness of whether a stimulus shows an object or an animal.

In the present study, the stimulus was a ring (annulus) that was shown in the middle of the screen. The ring comprised a Gabor patch tilting either left or right (${\pm }45^{\circ }$). To avoid confounding effects of task differences, subjects performed two tasks on each trial: a detection task and an identification task. Separate staircase procedures were administered concurrently to target the detection threshold or the identification threshold. For each staircase, opacity of the ring was adjusted over trials so that about half of the trials were reported as aware and the other half as unaware. On each trial, subjects rated their awareness on a three-level PAS: experiencing nothing (PAS1), experiencing something (PAS2), or experiencing the orientation of the grating (PAS3). Then, they rated whether the ring tilted left or right by choosing one of two circles (tilting left or right). Immediately after their response about the orientation of the grating, subjects received performance feedback. In the present study, the detection threshold (representing lower-level experiences) was defined as the difference between PAS2 and PAS1 (i.e., PAS2 − PAS1), and the identification threshold (representing higher-level experiences) was defined as the difference between PAS3 and PAS2 (i.e., PAS3 − PAS2).

PAS ratings were developed on the basis of a pilot study, as recommended (Sandberg & Overgaard, 2015). PAS3 was described as follows: The tilt is experienced to the degree that one believes that a subsequent response about the tilt (left or right) will be correct. Instructions emphasized that subjects should primarily report on their conscious experience (Sandberg et al., 2013; Sandberg & Overgaard, 2015). Performance feedback was provided only to help subjects anchor their experience. This secondary reference to performance and certainty was already included in the original PAS: PAS3 included *a feeling of almost being certain about one’s answer* and PAS4 included *no doubt in one’s answer* (Ramsøy & Overgaard, 2004). These definitions acknowledge that when subjects report that they are aware of a stimulus, they can typically identify the stimulus with certainty.

From the perspective of LoP (Jimenez et al., 2020; Windey et al., 2014), the present stimulus and tasks are low level. When designing the present study, we had to choose between low level or high level because even with only a single level, each subject took almost 90 min to complete the experiment. Because most of the previous evidence against the strong version of the claim comes from studies with a low-level stimulus/task (Derda et al., 2019; Jimenez et al., 2018; Tagliabue et al., 2016), it seemed most informative to examine the claim by Koivisto et al. (2017) in the context of a low-level stimulus/task.

In most previous studies, initial staircase procedures were used to adjust the stimulus to a level so that about half of the trials are reported as aware and the other half as unaware (i.e., awareness threshold). Then, the stimulus is held constant during the actual experiment. Although this approach avoids a physical stimulus confound (Aru et al., 2012), it has several drawbacks. First, subjects may be excluded because for these subjects, the relative percent of aware and unaware trials in the actual experiment differs too much from 50%; thus, stimuli were apparently not presented at the awareness threshold. For example, Koivisto et al. (2017) excluded 4 of 16 (25%) subjects, and we had to exclude 7 of 35 (20%) subjects in one session and 13 of 35 (37%) subjects in a second session in our previous study on VAN and LP (Eklund & Wiens, 2018). Second, a claim for an awareness threshold is often based on group data. That is, across subjects, maybe half of the trials are reported as aware, but this ignores how much individual subjects differ from this mean. For example, a group mean of 50% aware trials suggests that across subjects, half of the trials were rated as aware, but this may not apply to any actual subject: A subject may have 70% aware and 30% unaware trials (or vice versa), and the chosen stimulus intensity was apparently over (or under) the actual awareness threshold. Third, for a given subject, the proportion of aware and unaware trials is commonly analyzed across the whole experiment. This procedure is insensitive to changes in the awareness threshold during the experiment. For example, an individual subject might give mostly aware responses early in the experiment and mostly unaware responses later in the experiment. Across all trials, half of the trials might be rated as aware and half as unaware, but the data suggest that the awareness threshold changed over time.

To minimize these problems in the present study, stimulus opacity was adjusted continuously over trials to match the detection threshold and the identification threshold. During subsequent processing of each subject’s data for each threshold, trial blocks were identified in which the number of aware and unaware trials did not deviate from chance, and only these trials were analyzed further. The goal of this procedure was to ensure that the analyzed data corresponded closely to the concept of an awareness threshold (i.e., about 50% aware and unaware trials) both within subjects and between subjects. Then, we controlled for effects of physical differences in opacity by entering the variable of interest (awareness) in parallel with a potentially confounding variable (opacity) in the statistical model. This approach is recommended because the unique contribution of each variable is assessed (Sassenhagen & Alday, 2016).

Notably, keeping the stimulus constant assumes that physical differences must be avoided because they necessarily confound the results. However, this assumption seems unnecessarily strict, particularly so if the physical differences are tiny (Sassenhagen & Alday, 2016). For example, Koivisto et al. (2017) used four different digits (3, 4, 6, and 7) and did not report whether some digits were rated as aware more often than other digits. A critic may argue that if aware and unaware conditions differed in which digits were shown, it cannot be ruled out that there was a physical stimulus confound. Although this is true, it appears that previous studies have not controlled for these minor differences, apparently because these differences were not considered to be noteworthy. Importantly, the statistical approach allows one to test for a physical confound empirically by determining the contribution of physical differences as well as awareness effects (Alday & van Paridon, 2021; Sassenhagen & Alday, 2016). In the present analyses, stimulus opacity was included as a separate predictor to examine unique contributions of awareness on mean amplitudes (Kretzschmar & Alday, 2020; Sassenhagen & Alday, 2016).

In contrast to previous studies, we conducted Bayesian analyses (Dienes, 2008, 2016; Wagenmakers et al., 2016; Wiens & Nilsson, 2017) to measure the strength of evidence for or against the presence of each of three effects: an effect of awareness for lower-level experiences (detection), an effect of awareness for higher-level experiences (identification), and whether the effect of awareness differed between detection and identification (i.e., interaction between awareness and threshold). With regard to the strong interpretation (Koivisto et al., 2017), there should be evidence for no VAN for higher-level experiences (identification). With regard to the weak version of the claim, VAN should differ by threshold: VAN should decrease from higher-level experiences (identification) to lower-level experiences (detection). Although research has shown that LP is not an NCC, we report results for LP below because these are relevant to other aspects of consciousness (Dembski et al., 2021; Förster et al., 2020).

## Method

All material, data, and scripts are shared as online supplement via a public university repository (Wiens, 2023) to adhere to the recommendations of open science (Munafò et al., 2017).

*Participants*

We recruited 40 student volunteers. The advertisement stated that subjects should have normal or corrected vision (glasses or contact lenses), be between the age of 18 to 40, and have no mental health disorders or neurological history. However, we did not screen subjects for these criteria except age. The sampling plan was to recruit as many subjects as possible before the beginning of the Summer break in 2022. In accordance with local law and institutional requirements, ethical review and approval were not required for this study. The experiment adhered to the declaration of Helsinki. Participants provided their written informed consent to participate in this study and that their raw data will be shared anonymized. Participants received a 200 SEK gift voucher. As explained below, one subject was excluded because of too few EEG trials (*n* < 25) for both detection threshold and identification threshold. One subject was excluded because the subject did not do the tasks correctly. The remaining sample comprised 38 participants (16 male; 31 right-handed; age: *M* = 29.8, *SD* = 6.3).

As described below, subjects were excluded for either detection threshold or identification threshold if they did not have enough EEG trials (*n* < 25) after preprocessing of the EEG data. Of the sample of 38 subjects, 32 subjects provided data for both thresholds, 1 subject provided data only for the detection threshold, and 5 subjects provided data only for the identification threshold.

*Stimuli and apparatus*

The stimulus was of a 50-ms ring (annulus) with an inner diameter of 26 mm (2.6^∘^) and an outer diameter of 30 mm (3.0^∘^). The ring comprised a Gabor patch (spatial frequency = 20) that was tilted either left (-45^∘^) or right (+45^∘^). Grating opacity was adjusted to increase or decrease its visibility. Stimuli were shown on a 24-inch BenQ XL2430T monitor at 144 Hz with a resolution of 1,920 by 1,080. The experiment was performed in a dimly lit room. In-house scripts in PsychoPy (Peirce et al., 2019) were used to generate visual stimuli and to run the experiment.

**Fig. 1:**
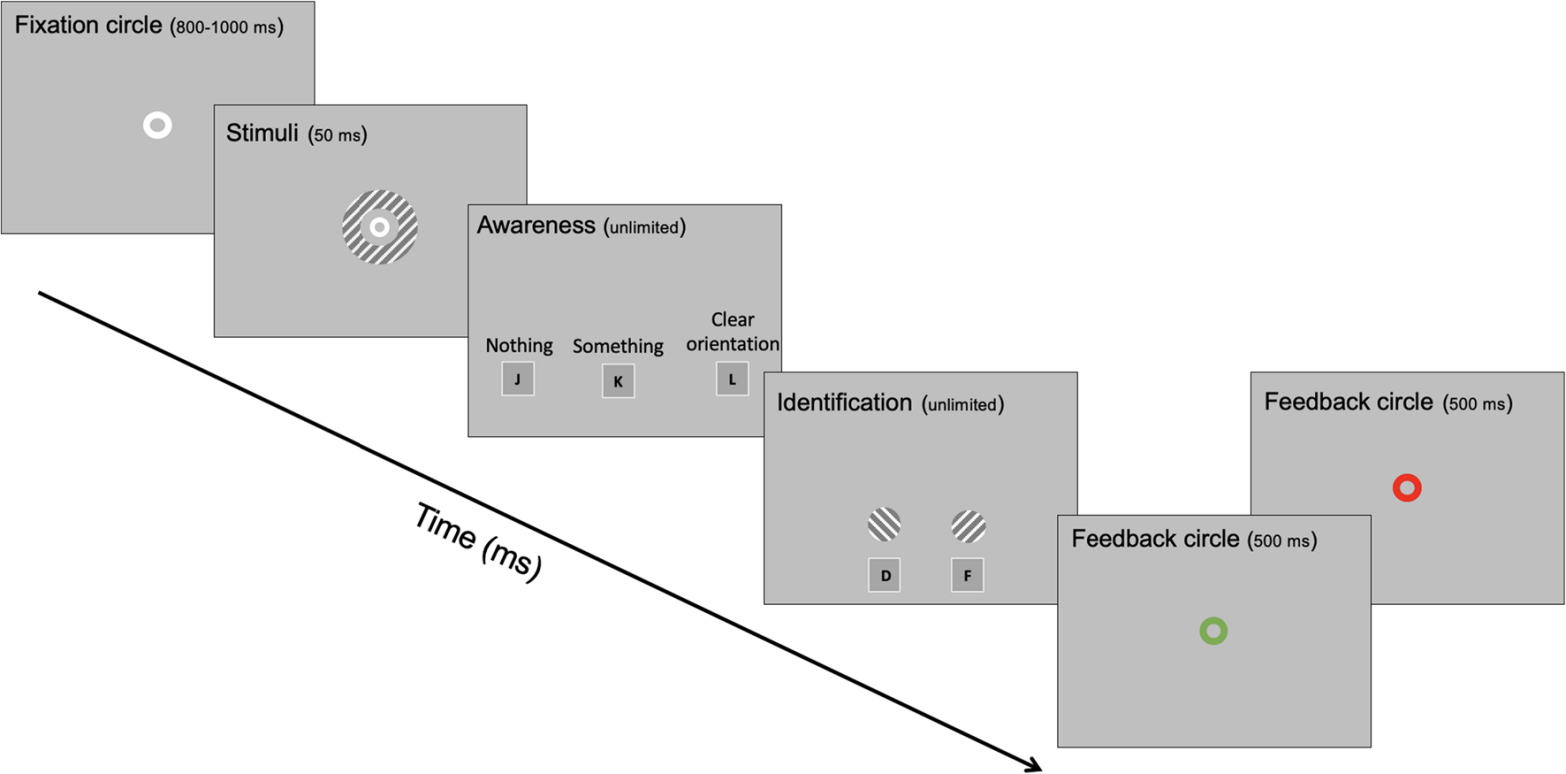
Time course of a trial. On each trial, a fixation circle was followed by a 50-ms presentation of a ring. Afterwards, subjects rated their subjective awareness of the ring: "Nothing", "Something," or "Clear orientation". Then, subjects reported the orientation of the grating (left or right). They received immediate feedback on whether their identification was correct (green) or incorrect (red)

*Procedure*

On each trial, subjects performed a detection task and an identification task while seated in front of the computer screen. Figure 1 shows the structure of a trial. Each trial started with a white fixation circle (0.7^∘^). After 800 to 1000 ms (randomly in steps of 50 ms), the ring was presented for 50 ms together with the fixation circle. Between 600 to 800 ms (randomly in steps of 50 ms) after the offset of the ring and fixation circle, an adapted PAS was shown and subjects rated their subjective awareness of the ring (Koivisto et al., 2017; Ramsøy & Overgaard, 2004; Sandberg & Overgaard, 2015). Response options for the PAS were nothing (PAS1), something (PAS2), and clear orientation (PAS3), as explained below. Then, subjects reported the orientation of the grating by choosing one of two schematic representations of the two orientations. Subjects received immediate performance feedback in that the subsequent fixation circle changed color for 500 ms (green for correct and red for incorrect). After the feedback, the next trial started immediately.

Critical trials (on which a grating was shown) varied in opacity level and direction of the grating. Opacity was determined by the specific staircase (detection or identification, see below), and direction of the grating was either left or right, resulting in 4 types of trials (plus catch trials). These types of trials were shown in pseudo-randomized order. To simplify the tasks and avoid memory confounds, relevant response alternatives and the corresponding buttons were shown on the screen until subjects responded. After each block, response hands were reversed and the mapping of stimuli to keys was changed; the goal was to avoid EEG confounds from consistent key-response mappings.

Subjects used a keyboard to respond. They rated awareness with one hand and orientation of the grating with the other hand. The left hand was used to rate PAS, and the right hand was used to report orientation, or vice versa. To rate PAS1, PAS2, and PAS3 with the left hand, buttons S, D, and F were used, respectively; and to rate PAS1, PAS2, and PAS3 with the right hand, buttons J, K, and L were used, respectively. To report orientation with the left hand, buttons D and F were used, and to report orientation with the right hand, buttons J and K were used. On each trial, subjects had to finalize each choice by pressing the space key. For each subject, mapping of response hands to the questions was randomly determined, and this mapping was reversed for each block. At the beginning of each block, the correct key-response mapping was shown on the screen to prepare subjects.

Before the main task, subjects familiarized themselves with the stimuli, the PAS ratings, and the reversal of the response hands. Subjects were presented with example trials of the ring and asked to describe their experience. Opacity of the ring was decreased over trials. Subjects’ experiences matched those of pilot subjects: *Nothing* (PAS1) implied having no experience of the visual stimulus, not even a faint sensation of something. *Something* (PAS2) implied having a weak sensation of something. This referred to experiencing any part of or the whole ring without being able to identify the orientation of the grating. *Clear orientation* (PAS3) implied experiencing (identifying) the orientation of the grating to the degree that subjects believed that the subsequent response about the orientation of the grating (left or right) would be correct.

These PAS ratings were chosen on the basis of a pilot study, as recommended (Sandberg & Overgaard, 2015). Pilot subjects were shown gratings that gradually decreased in opacity and subjects were asked to describe their experience after each grating. Subjects described their experiences in terms of nothing (PAS1), detecting something (PAS2), and identifying the orientation of the grating (PAS3). Although the clarity of the grating could be stronger (PAS4), subjects found that it was difficult to distinguish between PAS3 and PAS4. This difficulty shows that once the gratings were identified, the experience of the gratings increased gradually without any evidence for a threshold. Thus, results supported the use of only three PAS levels. At first, we thought it would be useful to include PAS4 anyway, but pilot subjects reported that retaining PAS4 was confusing because they used it only for a few trials at the very beginning of the staircase.

Initially, we planned to run the main task without performance feedback on the orientation of the grating. However, some pilot subjects answered PAS3 even though they were unable to identify the orientation of the gratings (i.e., performance was at chance, 50%), whereas others answered PAS2 even though they were able to identify the orientation of the gratings. To reduce these individual differences in identification thresholds, we introduced performance feedback on each trial (see Fig. 1). Thus, PAS3 was defined as the experience of the orientation of the grating to the degree that subjects believed that the subsequent response about the orientation of the grating (left or right) would be correct. When we asked subjects informally at the end of the experiment, many subjects reported that they found the performance feedback helpful in anchoring their conscious experiences. They also noted that the feedback helped them stay motivated throughout the experiment.

Subjects practiced until they reported that they understood the tasks (see Fig. 1). Instructions emphasized that the primary task was to rate conscious experiences accurately whereas identifying the orientation of the grating was secondary. With regard to performance feedback, they were instructed that for PAS3, their subsequent response about the orientation of the grating should be correct. For PAS1 and PAS2, they may guess orientation and should not worry about whether their response was correct. Further instructions were as follows: Subjects had to the press the space key to finalize their choice to minimize response errors. Subjects did not have to memorize the mapping between response alternatives and keys, as the relevant response alternatives and the corresponding keys would be shown until response. On subsequent blocks, response hands would alternate. The tasks consisted of blocks that lasted about 5 min each; subjects were encouraged to rest for a few minutes during the breaks.

Most subjects (ID thru 32, *n* = 29) performed an initial staircase procedure (Eklund & Wiens, 2018): Two interleaved staircases were used to adjust opacity to the detection threshold (PAS2 vs. PAS1) and to the identification threshold (PAS3 vs. PAS2). No EEG was recorded during this initial staircase. Opacity was adjusted depending on subjects’ responses on each trial. Each staircase comprised 50 trials (half with left orientation and half with right orientation), and step size decreased with each second reversal. To estimate the detection and identification thresholds, a psychometric response function (binomial with a probit link) was fitted to the data from each staircase (Wichmann & Hill, 2001). If the data did not suggest convergence for one or both thresholds, the staircase procedure was repeated (13 of 29 subjects had up to 4 staircases; mean = 2.4).

For early subjects (ID thru 32, *n* = 29), the main task (with EEG recording) comprised 400 critical trials and 20 catch trials. Trial order was pseudo-random in sections of 21 trials (10 left, 10 right, and 1 catch). Across critical trials, half of the trials targeted the detection threshold and the other half the identification threshold (in random order). The interleaved staircases adjusted opacity either according to the 1-up and 1-down rule (*n* = 16) or the 2-up and 2-down rule (*n* = 14). We switched to the 2-up and 2-down rule for later subjects because opacity would remain more stable and would not be changed on every trial.

For subsequent subjects (ID 33 thru 43, *n* = 11), the initial staircases without EEG recording were omitted to collect data efficiently. Thus, two interleaved staircases (2-up and 2-down rule) were administered while EEG was recorded. Subjects performed about 100 trials in between breaks for a total of about 500 trials. For each trial, the probability for a ring was 0.475 for left and 0.475 for right, and the probability for no ring (catch) was 0.05. For these later subjects, opacity settings and the timings for breaks could be adjusted online by the experimenter.

In the experiment software, opacity could be defined between 0 (transparent) and 1 (opaque). The number of steps (resolution) between 0 and 1 could also be defined. Over the course of testing subjects, we adjusted opacity resolution to improve the staircase procedure. Whereas for early subjects, opacity resolution was 500 for both detection and identification, for later subjects, we increased it to 200,000 for detection and 5000 for identification. A challenge in adjusting resolution was that if the chosen resolution was too low, the staircase would jump between two values (e.g., lower value = unaware and higher value = aware). In contrast, if the chosen resolution was too high, the staircase would require many trials to adjust opacity to the adequate level. Resolution values for individual subjects are reported in the supplement. We note that there is no theoretical reason why resolution differences between subjects should bias results; differences in resolution determined only whether changes in opacity between steps were relatively small or large. We also note that we chose these resolutions without knowing the highest resolution that could be physically resolved on the screen. That is, which changes in input opacity values would produce actual physical stimulus changes and which would not (e.g., input opacities of 0.00012 and 0.00013 may have the same opacity on the screen). After data collection was completed, we measured the relationship between input opacity values and actual changes in luminance on the screen (photodiode). We varied input data between 0 and 0.1 in 20,000 steps. We set 0.1 as the maximum because trials in the final analyses had opacities below this value. Also, we examined 20,000 steps because this corresponded to a maximum resolution of 200,000 used in the present study. Results showed that the software could not resolve 200,000 steps, but there was a nearly perfect relationship between input opacity and luminance (*r* =.99). In the actual analyses, we recoded input opacity so that the range of opacity values that produced identical luminance changes was represented by the mean opacity of the range, as further described in the supplement. As a result, individual recorded opacity values were associated with different physical changes. To facilitate interpretation, opacity levels for each subject were converted to a scale ranging from 0 (transparent) to 100 (opaque); thus, 100 corresponds to a value of 1 in the software.

*EEG recording*

EEG data were recorded from 64 electrodes at standard 10–20 positions with an Active Two BioSemi system (BioSemi, Amsterdam, Netherlands). An EEG cap (Electro-Cap International, Eaton, OH) was used to position these electrodes together with two system-specific electrodes. Data were sampled at 512 Hz and filtered with a hardware low-pass filter at 102.4 Hz.

*EEG preprocessing*

All scripts are provided in the online supplement (Wiens, 2023). The continuous EEG data from the 64 standard electrode positions were processed and analyzed offline with MNE-Python (Gramfort et al., 2013, 2014). In an initial processing step for each subject, noisy channels and eye blink components were identified. To that end, a 1-Hz high-pass filter and a 40-Hz low-pass filter were applied. Electrodes were visually inspected for excessive noise. Only a few noisy electrodes had to be interpolated (with spherical spline interpolation) from neighboring electrodes (*M* = 0.35, *SD* = 1.09). Then, independent component analysis (fastica) was conducted, and eye blink components were identified on the basis of their topography (*M* = 1.05, *SD* = 0.38). After this initial processing step, the raw data were read in again, bad channels were interpolated, data were average referenced, eye blink components were excluded, and a 0.1 high-pass filter was applied.

Epochs were extracted from 100 ms before to 800 ms after stimulus onset. Each epoch was baseline corrected to the mean of the 100-ms interval before stimulus onset (-100 to 0 ms). For each subject, maximum amplitude ranges were extracted for individual epochs (after a 30-Hz low-pass filter), and the distribution of these amplitude ranges was inspected for outliers. The number of identified outliers per subject was *M* = 18.62 (*SD* = 17.89), corresponding to *M* = 4.21% (*SD* = 3.69). The exclusion thresholds were set for each individual because subjects showed substantial variability in these amplitude ranges. Critically, to avoid bias, inspection of trials was blinded to trial type, PAS rating, and response about orientation (Keil et al., 2014). Finally, the epoched data were downsampled to 256 Hz.

VAN-relevant mean amplitudes were computed between 180 and 280 ms after ring onset across electrodes O1, O2, PO3, PO4, PO7, and PO8. LP-relevant mean amplitudes were computed between 350 and 550 ms after ring onset across electrodes Pz, P1, P2, CPz, CP1, and CP2. These intervals were identical to those in our previous study (Eklund & Wiens, 2018), but we included more electrodes than before. After exclusion of trials marked as bad during EEG preprocessing, the mean numbers of valid trials for the four conditions (unaware/aware by detection/identification) ranged between 63.4 (SD = 20.6) and 77.5 (22.3).

*Data analysis*

**Fig. 2:**
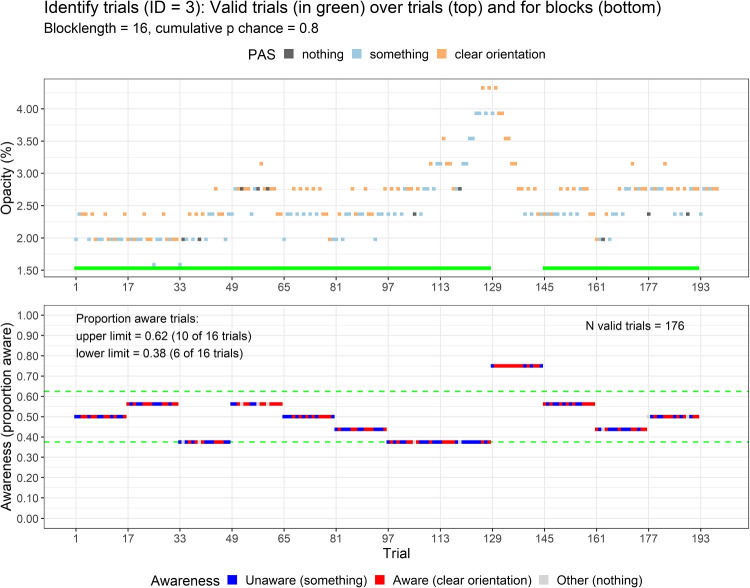
Opacity levels and PAS ratings for identification trials for an individual subject. The top panel shows opacity levels and PAS ratings over trials. The bottom panel shows the proportion of aware ratings in each block. Green denotes that the number of aware ratings was within chance (i.e., between 6 and 10 out of 16 trials)

After preprocessing of the EEG data in MNE-Python (Gramfort et al., 2013, 2014), subsequent data analyses were done in *R* using *Quarto* within *R Studio* (Allaire et al., 2021; Bürkner, 2017; Makowski et al., 2019; R core Team, 2016; RStudio Team, 2020; Wickham et al., 2019; Xie et al., 2019). All scripts for the main and additional analyses are reported in the online supplement (Wiens, 2023). For each subject, the behavioral data during EEG recording were processed separately for each staircase (detection and identification). For detection, PAS2 represented aware and PAS1 unaware, and for identification, PAS3 represented aware and PAS2 unaware. The consecutive trials in each staircase were divided into blocks of 16 trials. A block was considered to be at the awareness threshold if the proportion of aware trials was within a reasonable range expected by chance. For a block length of 16 trials, a binomial distribution suggested that between 6 and 10 aware trials would be expected by chance at a cumulative probability below.80. Accordingly, individual blocks were considered valid if the number of aware trials was between 6 and 10. Figure 2 illustrates this definition of valid blocks for an individual subject. One subject had an unreasonable response pattern: Even though opacity levels were much higher during identification than detection, the subject reported being unaware of many identification trials (at very high opacity) while also being aware on many detection trials (at very low opacity). This subject was excluded completely.

For each subject, only trials in valid blocks were processed further. For each block, opacity was mean centered to remove drift; thus, only relative differences in opacity within a block were considered in the analyses. Individual trials were merged with the preprocessed EEG data, which contained mean VAN-relevant and LP-relevant amplitudes and marked whether each trial was considered bad during preprocessing. In the analyses reported below, trials with bad EEG were excluded. If subjects had fewer than 25 trials in either unaware or aware condition for a threshold condition, they were excluded. Subsequently, individual trials were excluded separately for VAN and LP if the mean amplitude deviated more than 3 SDs from the mean across all trials and subjects (less than 1% of the remaining trials were removed for either VAN or LP). As described below, robustness checks showed that results were unaffected by different criteria of block length, chance level, and exclusion of bad EEG trials.

Bayesian robust mixed effects regression models were used to analyze mean amplitudes at VAN-relevant and LP-relevant mean amplitudes on trial-level data (Alday & van Paridon, 2021; Brown, 2021; Franke & Roettger, 2019; Kretzschmar & Alday, 2020). The data were not centered or standardized. Bayesian models were estimated using *brms* (Bürkner, 2017, 2018). In all models, vague priors were used for intercepts and slopes (i.e., normal distribution with *M* = 0 and *SD* = 2). Because mixed effects models include all available data, they reduce the risk for biased effect size estimates (Matta et al., 2018). Results of the models include mean estimates and Bayesian confidence intervals.

Bayesian hypothesis testing was conducted with Bayes factors in *bayestestR* (Makowski et al., 2019). Bayes factors compare different models (e.g., null vs. alternative hypothesis) and provide evidence for or against a particular model (Dienes, 2016; Wagenmakers et al., 2016; Wiens & Nilsson, 2017). Thus, Bayes factors avoid mistaking nonsignificance as evidence for no effect (Dienes, 2008; Makin & Orban de Xivry, 2019; Wasserstein & Lazar, 2016). The Bayes factor (*BF*) is a continuous measure of the relative evidence for one model versus another. For example, a *BF10* > 3 means that the data support the presence of an effect three times more than the absence of an effect, whereas a *BF01* > 3 suggests the opposite (Dienes, 2016). Although BF is a continuous measure of evidence, we use verbal labels to describe the strength of evidence (Wagenmakers et al., 2018).

The goal of the first analysis was to show that VAN and LP can be observed at the detection threshold. The analysis considered only the EEG data for the detection threshold and examined effects of awareness and opacity on mean amplitudes (i.e., VAN-relevant and LP-relevant mean amplitudes). Awareness (unaware vs. aware) and opacity (continuous variable) were modeled as fixed effects and were allowed to vary randomly across subjects (i.e., varying slopes and intercept over subjects). Awareness was dummy coded as 0 (unaware) and 1 (aware). Thus, awareness captured effects of awareness on mean amplitudes independent of opacity. The model formula in *brms* was as follows: EEG $\sim$ 1 + awareness + opacity + (1 + awareness + opacity | id).

The main, second analysis examined effects of awareness for both thresholds (identification and detection). Fixed effects included the interaction of awareness and threshold and the interaction of opacity and threshold (together with lower-order effects). These effects were allowed to vary randomly across subjects. Awareness was dummy coded as 0 (unaware) and 1 (aware), and threshold was dummy coded as 0 (identification) and 1 (detection). Thus, the effect of awareness captured effects of awareness for the identification threshold, and the interaction of awareness with threshold captured the change of the awareness effect from identification to detection. The model formula in *brms* was as follows: EEG $\sim$ 1 + threshold * awareness + threshold * opacity + (1 + threshold * awareness + threshold * opacity | id). Note that this model automatically includes lower-order effects (i.e., threshold, awareness, and opacity).

Finally, a simplified multiverse analysis was conducted to examine the robustness of the present results (Steegen et al., 2016). To that end, 16 analyses were conducted that differed in their analysis settings: First, block length was either 16 or 20. Second, chance level for a block was either liberal or conservative. For a block length of 16, the liberal criterion (*p* <.80) permitted between 6 and 10 aware trials, and the conservative criterion (*p* <.55) permitted between 7 and 9 aware trials. For a block length of 20, the liberal criterion (*p* <.75) permitted between 8 and 12 aware trials, and the conservative criterion (*p* <.50) permitted between 9 and 11 aware trials. Third, trials marked as bad during EEG preprocessing were either included or excluded. Fourth, orientation of the Gabor (left or right) was either included or excluded as an additional predictor in the mixed models. Thus, this criterion assessed whether orientation of the Gabor had any effects on VAN and LP. These four criteria yielded 16 combinations of analysis settings. We limited analyses to these 16 combinations because we deemed it unfeasible and unnecessary to run more analyses (each analysis took about 2 h to complete).

Even though opacity could vary between 0 and 100, opacity levels were relatively low in all conditions. Across subjects, the raw means for each condition were 0.95 (*SD* = 0.34) for unaware detect, 1.16 (*SD* = 0.31) for aware detect, 2.80 (*SD* = 1.24) for unaware identify, and 2.90 (*SD* = 1.24) for aware identify. Despite the tiny differences between unaware and aware in each threshold, opacity was included as a predictor in the main analyses to remove its potentially confounding effect on mean amplitudes (Sassenhagen & Alday, 2016).

## Results

*Behavior*

Performance in correctly reporting the orientation of the grating (left or right) varied over conditions. The first Bayesian logistic mixed model examined whether the proportion of correct responses differed between unaware and aware trials in the detection task. Results suggested that the estimated proportion correct was similar for unaware detection (*M* = 0.51) and aware detection (*M* = 0.53), *BF01* = 13. The second Bayesian logistic mixed model included awareness (unaware, aware) and threshold (identification, detection). With regard to identification, results suggested that the estimated proportion correct was larger for aware identification (*M* = 0.97) than unaware identification (*M* = 0.72), *BF10* $> 1.9 * 10^{10}$. These behavioral findings validate the use of PAS in our study: Subjects had lower performance during detection than identification.

Further, we note that higher performance for unaware identification (72%) than aware detection (53%) is not surprising. In both cases, subjects responded PAS2, but the definition of PAS2 covered a range of experiences (see Method section). For the detection threshold, PAS2 mainly captured experiences of a weak sensation of something (compared to nothing). Thus, performance was low for aware trials (PAS2). For the identification threshold, PAS2 mainly captured experiences of any part of or the whole ring without being able to identify the orientation of the grating (compared to experiencing the orientation). Accordingly, performance was relatively high during unaware trials (PAS2) and excellent during aware trials (PAS3). Also, stimulus opacity was generally stronger on PAS2 trials during identification (2.80) than on PAS2 trials during detection (1.16), as reported earlier. Taken together, these findings support the conclusion that PAS2 was used to capture different experiences during identification and detection.

Catch trials of early subjects could not be used because of a software bug (i.e., the grating was visible). For the remaining 25 subjects, subjects received an average of 22.7 catch trials (*SD* = 4.4); they were unlikely to make a false alarm by responding PAS2 or PAS3 (*M* = 0.9, *SD* = 1.6).

*EEG*

**Fig. 3:**
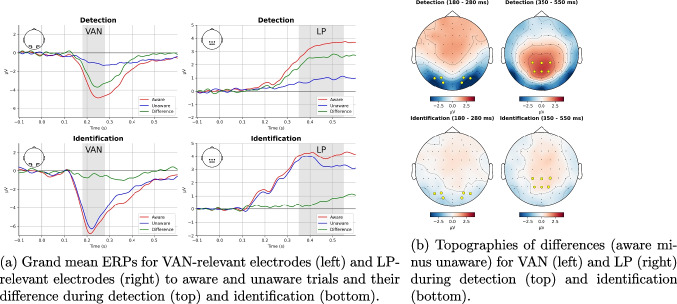
Grand mean ERPs (left) and topographies (right) for VAN-relevant and LP-relevant amplitudes

**Fig. 4:**
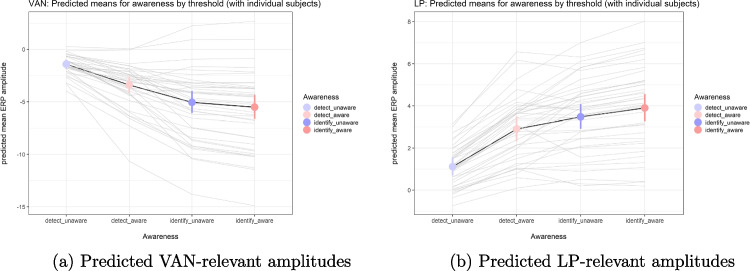
Predicted mean amplitudes (and individual 95%CI)

Figure 3a shows grand mean ERPs for VAN-relevant and LP-relevant amplitudes during detection and identification. Figure 3b shows topographies of the difference between aware and unaware for VAN and LP during detection and identification. Both figures support the choice of intervals and electrodes, similar to that in our previous study (Eklund & Wiens, 2018). Figure 4 shows the predicted means for VAN-relevant amplitudes (left panel) and LP-relevant amplitudes (right panel), as estimated by a model that includes the interaction of awareness and threshold.

As confirmed by statistical analyses reported below, the figures show that during detection, there was extreme evidence for VAN: Amplitudes were more negative during aware than unaware trials. However, there was ambiguous support for VAN during identification. Further, LP was present during both detection and identification: Amplitudes were more positive during aware than unaware trials. Critically, results showed that effects of awareness on mean amplitudes were larger during detection than identification. Accordingly, VAN and LP decreased strongly from detection to identification.

For VAN-relevant amplitudes (see Fig. 4, left), the first Bayesian robust linear mixed model of the detection data showed that amplitudes were more negative to aware than unaware; mean = $-$2.12 $\mu$V, 95%CI [$-$2.86, $-$1.42], *BF10* > 188,000. This finding provides extreme support for VAN. Also, the data provided extreme support for an effect of opacity; mean = $-$3.61 $\mu$V, 95%CI [$-$5.12, $-$1.99], *BF10* = 498. The second model of the identification and detection data showed that there was ambiguous support for VAN during identification; for the difference of aware minus unaware, mean = $-$0.46 $\mu$V, 95%CI [$-$0.79, $-$0.11], *BF10* = 2.3. Because the BF was ambiguous, we also analyzed other Bayesian indices (Makowski et al., 2019). For the main data set, probability of direction (pd) = 99.4%, and given a region of practical equivalence (ROPE) between $-$0.1 and +0.1 $\mu$V, ROPE(full) = 2%. In the second model, there was moderate support against an effect of opacity; mean = 0.04 $\mu$V, 95%CI [$-$0.91, 0.99], *BF01* = 4.3. Critically, there was extreme support that VAN was larger (more negative) during detection than identification; mean difference = $-$1.51 $\mu$V, 95%CI [$-$2.20, $-$0.77], *BF10* = 131. Also, there was extreme support that the effect of opacity varied by task; mean = $-$3.73 $\mu$V, 95%CI [$-$5.34, $-$2.02], *BF10* = 594. Note that scripts and results for this and all other analyses can be found in the online supplement (Wiens, 2023).

**Fig. 5:**
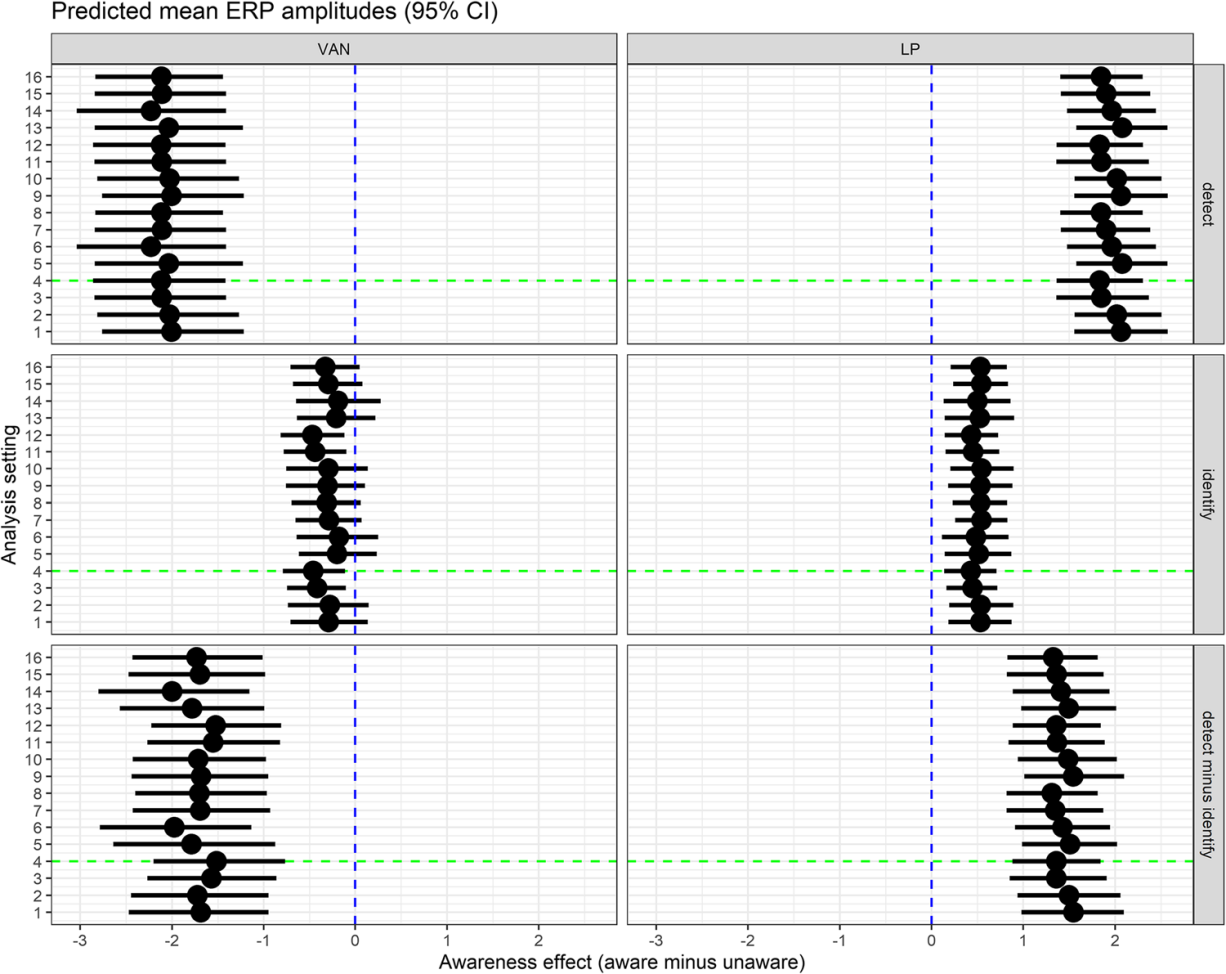
Predicted mean amplitudes (and 95%CI) for VAN and LP (i.e., aware - unaware) during detection (top), identification (middle), and the difference between detection and identification (bottom) for 16 different analysis settings. Note: The green line marks the model with the analysis settings reported in the main text. The term detect - identify refers to the difference in VAN or LP between detection and identification

For LP-relevant amplitudes (see Fig. 4, right), the first Bayesian robust linear mixed model of the detection data showed that mean amplitudes were more positive during aware than unaware; mean = 1.83 $\mu$V, 95%CI [1.36, 2.30], *BF10* > 260,000. This finding provides extreme support for an LP. Also, the data provided extreme support for an effect of opacity; mean = 3.19 $\mu$V, 95%CI [1.92, 4.44], *BF10* = 1212. The second model of the identification and detection data showed that there was moderate support for LP during identification; for the difference of aware minus unaware, mean = 0.43 $\mu$V, 95%CI [0.14, 0.71], *BF10* = 3.83. There was ambiguous support for an effect of opacity; mean = 0.73 $\mu$V, 95%CI [$-$0.04, 1.49], *BF10* = 1.12. Critically, there was extreme support that LP was larger (more positive) during detection than identification. Specifically, the awareness difference (aware minus unaware) was more positive during detection than identification; mean = 1.36 $\mu$V, 95%CI [0.88, 1.84], *BF10* = 3808. There was very strong support that the effect of opacity varied by task; mean = 2.43 $\mu$V, 95%CI [1.00, 3.85], *BF10* = 80.

Figure 5 shows results of the multiverse analysis. That is, results of 16 analyses that differed in their analysis settings in terms of block length, definition of chance level, exclusion of EEG trials marked as bad during preprocessing, and inclusion of orientation of the Gabor as an additional predictor. For each analysis setting, the figure shows predicted mean amplitudes (and 95%CI) for VAN and LP for detection and identification and also for the interaction (i.e., whether effect of awareness differed between detection and identification). Figure 5 suggests that results were robust to differences in analysis settings.

## Discussion

Results of Bayesian analyses (i.e., Bayes Factors) provided extreme evidence that VAN and LP for lower-level experiences (detection) were larger than VAN and LP for higher-level experiences (identification). For identification, the BF was ambiguous but other Bayesian indices provided rather strong support for the presence of VAN. Evidence for the presence of LP was moderate. These findings demonstrate that VAN and LP are more sensitive to the experience of seeing something (detection) than to the experience of identifying what was shown (identification).

Koivisto et al. (2017) pointed out that when studying NCC, one needs to consider the level of the experience: lower-level experiences associated with detection and higher-level experiences associated with identification. In their study, the original PAS was modified to distinguish between experiencing something without being able to identify the stimulus (PAS2) and experiencing the stimulus and being able to identify it (PAS3). Results showed that VAN was larger at the detection threshold (PAS2 − PAS1) during a detection task than at the identification threshold (PAS3 − PAS2). There was no significant VAN at the identification threshold. Results suggested that LP was unaffected by task. The authors claimed that VAN is an NCC of lower-level experiences (detection) but not of higher-level experiences (identification).

This claim can be interpreted in two versions: A *strong* version is that there is no VAN for higher-level experiences (identification). A *weak* version is that VAN is larger (more negative) for lower-level experiences (detection) than for higher-level experiences (identification). Because in the original study (Koivisto et al., 2017), detection and identification were measured during different tasks with different instructions, results may be confounded by task differences. The present study avoided this issue by measuring both detection and identification simultaneously. Also, the present study retained the critical features of defining PAS with reference to detection and identification and of showing low-contrast stimuli at the center of the screen (Koivisto et al., 2017).

Bayesian analyses of the present data provided extreme evidence that VAN and LP are larger for detection than identification. As such, the present findings replicate and extend results of previous studies. They replicate results that VAN is larger for detection than identification (Koivisto et al., 2017). Critically, they extend previous results because a potential task confound was avoided. Also, the present results show that the effect is similar for LP as for VAN: LP is larger for detection than identification. Whereas other studies have reported a non-significant difference between consecutive PAS ratings (Derda et al., 2019) or have not tested the relevant comparison explicitly (Jimenez et al., 2018; Koivisto et al., 2017; Tagliabue et al., 2016), the present Bayesian analyses provide extreme evidence that VAN (and LP) are more sensitive to lower-level experiences (detection) than higher-level experiences (identification).

Other relevant EEG studies reported findings that seem inconsistent with either version of the claim (Derda et al., 2019; Jimenez et al., 2018, 2021; Tagliabue et al., 2016). Most of these studies measured effects of PAS separately for a low-level stimulus/task and a high-level stimulus/task. Because the claim by Koivisto et al. (2017) is not specific to a particular stimulus, differences between low-level experiences (detection) and high-level experiences (identification) for a low-level stimulus (e.g., color) and a high-level stimulus (e.g., digit) can be viewed as separate tests of the claim. Previous studies reported that VAN-relevant amplitudes became increasingly more negative with PAS ratings for a low-level stimulus (Derda et al., 2019; Jimenez et al., 2018; Tagliabue et al., 2016) and a high-level stimulus (Derda et al., 2019). Similarly, LP-relevant amplitudes became increasingly more positive with PAS ratings for a low-level stimulus (Derda et al., 2019; Tagliabue et al., 2016) and a high-level stimulus (Derda et al., 2019; Jimenez et al., 2021). Because these findings suggest VAN for higher-level experiences (identification), they do not appear consistent with the claim by Koivisto et al. (2017) and with the observed decrease in VAN in the present study. However, there may be no contradiction if it is assumed that in previous studies, subjects used ratings of PAS2, PAS3, and PAS4 to refer to a gradual increase in visibility. If previous subjects did not consistently interpret PAS3 (or PAS4) as a qualitatively different experience in terms of identification, previous studies might have missed the decrease in VAN and LP for identification.

However, a critic may argue that in the present study, VAN to identification was reduced (if not eliminated) because of a ceiling effect. If VAN-relevant amplitudes at identification were already maxed out to unaware trials (PAS2), then VAN-relevant amplitudes could not have increased more to aware trials (PAS3). As shown in Figs. 3a and 4a, mean amplitudes at the VAN-relevant intervals were more negative for a stronger stimulus (i.e., unaware trials at identification) than for a weaker stimulus (i.e., unaware trials at detection). However, opacity levels at the identification threshold were generally low; on a scale between 0 and 100, mean opacity was 2.80 for unaware trials (PAS2) and 2.90 for aware trials (PAS3). Also, for identification, mean performance in correctly reporting the orientation of the grating was only 72% on unaware trials (PAS2). Because these observations suggest that stimuli at identification were relatively weak, they argue against the idea of a ceiling effect.

In the present study, subjects had to detect a ring and identify its orientation (left or right). This stimulus and task are considered low level by LoP (Jimenez et al., 2021; Windey et al., 2014). We used a low-level stimulus/task because most of the results that seemed inconsistent with the claim by Koivisto et al. (2017) used a low-level stimulus/task (Derda et al., 2019; Jimenez et al., 2018; Tagliabue et al., 2016). LoP predicts that for a low-level stimulus/task, conscious experiences are gradual, whereas for a high-level stimulus/task, conscious experiences are dichotomous (Windey et al., 2014). Importantly, this distinction does not seem relevant for the present study that focused on differences between two thresholds for the same stimulus. Accordingly, there is no *a priori* reason why the present findings for VAN would not generalize to a high-level stimulus (e.g., digits or letters).

The strong version of the claim by Koivisto et al. (2017) is that there is no VAN at the identification threshold (PAS3 − PAS2). Support for the absence of an effect is difficult to obtain with null hypothesis significance testing (Dienes, 2008; Makin & Orban de Xivry, 2019; Wasserstein & Lazar, 2016), particularly so if statistical power is low because of small sample size. Because sample size was 12 in the study by Koivisto et al. (2017), a non-significant VAN is uninformative. In the present study, we used Bayesian analyses to measure the evidence for or against an effect (Dienes, 2016; Makowski et al., 2019; Wagenmakers et al., 2016; Wiens & Nilsson, 2017). The BF provided ambiguous support for VAN at the identification threshold (*BF10* = 2.3); there was about as much evidence against as for the presence of VAN. Also, as shown in the middle left panel of Fig. 5, many 95%CIs for VAN at identification overlapped zero. Strictly speaking, these results do not resolve whether there is VAN at identification. Although it would have been possible to continue data collection until the BF is strong enough (in either direction), it was not feasible to do so in the present study. Furthermore, the BF is only one of various valuable Bayesian indices (Makowski et al., 2019). For the present study, these other Bayesian indices provide rather strong support for VAN at identification (Wiens, 2023). First, the probability of direction (pd) is defined as the proportion of the posterior that is in the same direction as the median of the posterior. For the main data set, pd = 99.4%; this means that the probability for a negative difference score (i.e., VAN) at identification is almost 100%. Second, the region of practical equivalence (ROPE) may be used to define a region within which effect sizes are considered to be practically equivalent to zero. When we (arbitrarily) defined ROPE to range between $-$0.1 and +0.1 $\mu$V, less than 2% of the posterior distribution fell within this ROPE. This means that the probability for a negligible effect size was very low. With regard to LP, results provided moderate support for LP at identification (*BF10* = 3.8). Thus, the BF suggested that there was three times more evidence for than against the presence of LP, but estimated LP was small. As shown in the middle right panel of Fig. 5, results of the same data with 16 different analysis settings confirmed that the findings for LP were robust. Taken together, for identification, BF results provided only ambiguous evidence for VAN and moderate evidence for LP. However, other Bayesian indices (pd, ROPE) provided rather strong support for VAN at identification.

Results from several older studies appear consistent with the idea of VAN for higher-level experiences (Koivisto & Revonsuo, 2008; Koivisto et al., 2005, 2009; Wilenius & Revonsuo, 2007). In a study of low-contrast line drawings (Wilenius & Revonsuo, 2007), a secondary analysis (*N* = 10) yielded VAN to trials when subjects reported to be very or absolutely sure about the content of objects compared with trials when subjects reported not be sure. Similarly, Koivisto et al. (2005) showed backward-masked letters (H, T, or U), and for each block of trials, one of the letters was defined as the target. A secondary analysis (*N* = 8) with constant masking conditions yielded VAN for aware target trials (hits) compared with unaware target trials (misses). Other, similar studies also reported VAN to masked, lateralized letters (*N* = 10, Koivisto et al. 2009) and masked gratings (*N*= 13, Koivisto and Revonsuo 2008). Unfortunately, all of these results may be confounded. First, if PAS1 trials (nothing) and PAS2 trials (something) are grouped together as unaware trials (e.g., Wilenius and Revonsuo, 2007), then the difference of aware minus unaware (i.e., PAS1 and PAS2 combined) does not isolate effects of higher-level experiences. For example, imagine that mean amplitudes are 0 $\mu$V for PAS1 trials, $-$0.2 $\mu$V for PAS2 trials, and $-$0.2 $\mu$V for PAS3 trials. Although there is no VAN at identification (i.e., PAS3 − PAS2 = 0), the difference of PAS3 trials minus the combined PAS1 and PAS2 trials would be $-$0.1. Critically, this difference would be completely driven by VAN to detection. Second, it may be that as soon as subjects can detect a target, they can identify it. If so, the difference between aware target trials and unaware target trials would be a combination of detection as well as identification. Importantly, to isolate effects of higher-level experiences, a critical design feature is that subjects may rate their experiences in terms of nothing (PAS1), lower-level experiences (PAS2), and higher-level experiences (PAS3), as in the present study. When testing for VAN for higher-level experiences, PAS3 trials have to be compared only with PAS2 trials (while PAS1 trials are excluded). Notably, a study using magnetoencephalography (MEG) showed rectangles that differed in orientation (Andersen et al., 2016). Results showed that activity in occipital sources in the VAN range differed between PAS2 and PAS3. Although these results were obtained with MEG, it is reasonable to assume that in studies with the critical design feature, VAN should be obtained for higher-level experiences. In fact, in the present study, whereas BF results were ambiguous, results of other Bayesian indices (pd, ROPE) provided rather strong support for VAN at identification.

In the present study, BF results provided extreme evidence for LP at lower-level experiences (detection) and moderate evidence for LP at higher-level experiences (identification). Further, BFs provided extreme evidence that LP decreased from detection to identification. Although these results resembled those for VAN, they do not imply that LP is an NCC. In the last decade, many studies in vision have shown that LP lacks sensitivity: Even though subjects are aware of the stimuli, LP is absent (Cohen et al., 2020; Dellert et al., 2021, 2022; Förster et al., 2020; Koivisto & Revonsuo, 2010; Kronemer et al., 2022; Pitts et al., 2012; Schlossmacher et al., 2020). Thus, the present results likely indicate that LP was present because of the task relevance of the stimuli. As shown in Fig. 4 (right), LP-relevant amplitudes were lowest when subjects reported seeing nothing (i.e., unaware detection) and increased when subjects were aware of the task-relevant stimuli. These findings suggest that LP-relevant amplitudes are sensitive to task-relevant stimuli that are detected. Also, the present findings provide convincing evidence that LP decreases from detection to identification when confounding effects of task differences are avoided. Thus, the present results extend previous studies that either did not address the present question or reported non-significant findings, which are not diagnostic (Derda et al., 2019; Jimenez et al., 2021; Koivisto et al., 2017; Tagliabue et al., 2016). From the LoP perspective, an interesting question is whether LP to identification might be larger for a high-level stimulus/task than for a low-level stimulus/task as used here. In support, two studies found larger LP for a high-level stimulus/task than a low-level stimulus/task (Derda et al., 2019; Jimenez et al., 2021) and one study reported no statistically significant difference (Jimenez et al., 2018). However, because it is unclear whether these studies specifically targeted higher-level experiences in terms of identification, it is unresolved whether these findings can be generalized.

Finally, we discuss two particular features of the present study. First, EEG data were recorded during staircase procedures that adjusted opacity over trials to target lower-level experiences (detection) and higher-level experiences (identification). During preprocessing of each subject’s data, trials were analyzed in blocks, and trials were retained only if the number of aware trials within a block did not deviate from chance. The goal of this approach was to ensure that the analyzed data matched the concept of an awareness threshold (i.e., similar numbers of aware and unaware trials) both within subjects and between subjects. We controlled for differences in opacity by including opacity as a separate predictor in the statistical model. This approach is common in linguistics because when using different linguistic stimuli, it is often impossible to avoid stimulus differences (Sassenhagen & Alday, 2016; Winter, 2019). Rather than assuming that stimulus differences must be avoided at all cost, the statistical approach determines whether stimulus differences affect the outcome variable. Recommendations are to try to keep the stimulus differences at a minimum and to estimate their effects on the outcome variable by modeling them explicitly (Sassenhagen & Alday, 2016). In the present study, opacity differences between aware and unaware trials were small. Nonetheless, opacity and awareness were modeled as separate predictors to estimate their unique contribution. In all statistical models, results showed that awareness affected VAN-relevant and LP-relevant amplitudes independently from opacity (Fig. 5).

Second, we reanalyzed the data with 16 different analysis settings to examine the robustness of the present results. Similar to fMRI data, EEG data can be preprocessed and analyzed in many different ways, and many of these different ways are equally valid (Carp, 2012; Keil et al., 2014). However, because EEG involves many preprocessing steps, it is often unresolved how robust the reported results are to the chosen preprocessing steps (Trübutschek et al., 2022). For researchers, the chosen analytic path may seem adequate to the data, but if analytic decisions are not made independently from the actual data, the risk for false positives increases strongly (Gelman & Loken, 2013, 2014; Luck & Gaspelin, 2017). Preregistration is a preferred method to assure other researchers that theoretically, results should be unbiased because preprocessing steps and analyses were chosen without any prior knowledge of the data (Nosek et al., 2018). In lieu of preregistration, we conducted 16 reanalyses of the data by varying several settings that we considered to be obvious candidates in affecting EEG results. Accordingly, we varied block length, chance level for a block, inclusion of outlier EEG trials, and inclusion of Gabor orientation in the statistical models. We did not vary the choice of electrodes and intervals. However, Figs. 3a and 3b suggest that the choice of intervals and electrodes captured VAN and LP well without overfitting (Keil et al., 2014; Luck, 2014). More advanced robustness checks are available, such as specification curve analysis, but these often require thousands of analyses (Simonsohn et al., 2020). Computing numerous models is feasible for frequentist analyses (that take several seconds) but not for the Bayesian analyses that we used, which took about 2 h per analysis. In sum, we varied relevant analytic settings to examine the robustness of the present results. Even though the number of alternative analyses was limited for practical reasons, their results attest to the robustness of the present findings.

Taken together, the present findings are important for recurrent processing theory (Lamme, 2006, 2010). A central tenant of this theory is that phenomenal consciousness is mediated by early, recurrent processing in sensory areas. Because VAN occurs relatively early and is generated in sensory areas (Dembski et al., 2021; Meyer, 2011; Snyder et al., 2015), VAN has been treated as an index of this processing (Eklund & Wiens, 2018; Förster et al., 2020; Lamme, 2018). In support, the present results showed extreme evidence for VAN for lower-level experiences (detection) and some evidence for VAN for higher-level experiences (identification). Thus, the present results are consistent with the idea that phenomenal consciousness is captured by VAN. However, because VAN was larger (more negative) for detection than identification, results suggest that VAN is more sensitive to lower-level experiences than higher-level experiences. However, even if VAN is not an ideal NCC of higher-level experiences, neural activity in relevant sensory areas has been shown to be an NCC. For example, when source activity in occipital areas was extracted from magnetoencephalography, machine learning could classify correctly between consecutive PAS ratings (Andersen et al., 2016). In sum, the present results emphasize that when searching for NCC, it is important to consider the level of experience and that a particular measure such as the VAN may be particularly sensitive to a particular level of experience.

In sum, Bayesian analyses provided extreme evidence that VAN is larger for detection than identification. These findings demonstrate that as an NCC, VAN is more sensitive to lower-level experiences of seeing something (detection) than to higher-level experiences of specific properties of the stimuli (identification). Consistent with the idea that lower-level experiences are generated in sensory cortices, VAN was strongest to these lower-level experiences. Thus, these results are consistent with recurrent processing theory in that phenomenal visual consciousness is reflected by VAN. Further, results emphasize that it is important to consider the level of experience when searching for NCC.

Open Practices Statement: The data and materials for the experiment are available via a public university repository (Wiens, 2023). During review, the link is private. After acceptance, the link will be available at this 10.17045/sthlmuni.21354195.

## Data Availability

All material, scripts, and data are available at a public university repository (Wiens, 2023)

## Code Availability

Scripts are available at a public university repository (Wiens, 2023)
